# On the effect of antiresorptive drugs on the bone remodeling of the mandible after dental implantation: a mathematical model

**DOI:** 10.1038/s41598-021-82502-y

**Published:** 2021-02-02

**Authors:** Mehran Ashrafi, Farzan Ghalichi, Behnam Mirzakouchaki, Manuel Doblare

**Affiliations:** 1 Faculty of Biomedical Engineering, Sahand University of Technology, Sahand New Town, Tabriz, Iran; 2 Tabriz Dental School, Orthodontic Department, Tabriz University of Medical Sciences, Tabriz, Iran; 3 Aragón Institute of Engineering Research (I3A), University of Zaragoza; Aragón Institute of Health Research (IIS-Aragón); Centro de Investigación Biomédica en Red en Bioingeniería, Biomateriales y Nanomedicina (CIBER-BBN), R&D Building, Block 5, 1st floor, Campus Rio Ebro, Mariano Esquillor s/n, 50018 Zaragoza, Spain

**Keywords:** Computational biophysics, Translational research

## Abstract

Bone remodeling identifies the process of permanent bone change with new bone formation and old bone resorption. Understanding this process is essential in many applications, such as optimizing the treatment of diseases like osteoporosis, maintaining bone density in long-term periods of disuse, or assessing the long-term evolution of the bone surrounding prostheses after implantation. A particular case of study is the bone remodeling process after dental implantation. Despite the overall success of this type of implants, the increasing life expectancy in developed countries has boosted the demand for dental implants in patients with osteoporosis. Although several studies demonstrate a high success rate of dental implants in osteoporotic patients, it is also known that the healing time and the failure rate increase, necessitating the adoption of pharmacological measures to improve bone quality in those patients. However, the general efficacy of these antiresorptive drugs for osteoporotic patients is still controversial, requiring more experimental and clinical studies. In this work, we investigate the effect of different doses of several drugs, used nowadays in osteoporotic patients, on the evolution of bone density after dental implantation. With this aim, we use a pharmacokinetic–pharmacodynamic (PK/PD) mathematical model that includes the effect of antiresorptive drugs on the RANK/RANK-L/OPG pathway, as well as the mechano-chemical coupling with external mechanical loads. This mechano-PK/PD model is then used to analyze the evolution of bone in normal and osteoporotic mandibles after dental implantation with different drug dosages. We show that using antiresorptive agents such as bisphosphonates or denosumab increases bone density and the associated mechanical properties, but at the same time, it also increases bone brittleness. We conclude that, despite the many limitations of these very complex models, the one presented here is capable of predicting qualitatively the evolution of some of the main biological and chemical variables associated with the process of bone remodeling in patients receiving drugs for osteoporosis, so it could be used to optimize dental implant design and coating for osteoporotic patients, as well as the drug dosage protocol for patient-specific treatments.

## Introduction

Bone remodeling is a biological process that develops in bone tissue throughout its whole lifetime. It denotes the process of new bone formation and old bone resorption that continuously modifies the internal microstructure and composition of bone. The main results of bone remodeling are: (i) to repair the internal damage generated by small-amplitude loads; (ii) to adapt the bone stiffness and strength to the specific mechanical demand; and (iii) to control the calcium equilibrium in the skeleton^1,2^. During the first stage of bone remodeling, old bone is removed (with its internal cracks) by the osteoclasts. These cells are activated by the osteocytes, as the cells responsible for detecting the environmental signals (e.g., strains, fluid flow, change in concentration or gradients of biochemical substances, etc.). This is followed by the second stage of new bone production performed by the osteoblasts that fill the areas previously resorbed by the osteoclasts^1^.

Understanding this process is important in many applications, such as optimizing the treatment of diseases like osteoporosis, maintaining bone density in extreme situations like microgravity or long-term periods of disuse, or assessing the long-term evolution of the bone surrounding prostheses after implantation. In particular, osteoporosis in the elderly (men and women) and especially in post-menopausal women, is highly prevalent^3^. A reduction in physical activity or the use of drugs such as steroids may promote excessive bone resorption, accelerating osteoporosis. This disease might accelerate the reduction in bone quality after implantation of osteosynthesis devices, joint prostheses or dental implants, increasing the probability of bone fracture^4,5^. Finally, it may cause a reduction in calcium concentration below its physiological level, which may promote other diseases^6^. Consequently, the treatment of osteoporosis with antiresorptive drugs such as bisphosphonates or denosumab is widely used^7,8^.

A particular case study of the effect of these drugs on the osteoporotic bone is the one of dental implantation. The increasing life expectancy in developed countries has boosted the demand for dental implants in patients with osteoporosis^9^. Several review studies^4,10,11^ conclude that bone healing time increases in osteoporotic patients which may endanger the success of dental implantation^12^. Histomorphological studies on bone evolution around Titanium implants in tibia^13^, in animal models with induced osteoporosis^14,15^, indicated that this disease leads to slower bone turnover and poorer bone-implant adhesion, which promotes reductions in the stiffness and strength of the bone-implant interface, which may drive to low trabecular bone density. Also, it has been repeatedly demonstrated that the failure rate of dental prostheses and implants, as well as the associated orthopedic equipment, increases when treating osteoporotic or low-quality bone^16^. Despite all this, there is not enough evidence to ban dental implants in osteoporotic patients, although a deeper study and additional improvements are required.

Several methods have been proposed to improve the stability of dental implants in patients with osteoporosis, including modifications in the implant design^17^, in the implant surface^18,19^, less invasive surgical techniques and complementary medical treatment. Drugs like bisphosphonates and denosumab are usually used to treat osteoporosis^20^, despite that their long-term use or a high dose may cause osteonecrosis^21–23^. These antiresorptive agents decrease osteoclast activity, thus reducing bone resorption, but simultaneously, they also reduce the bone remodeling rate, which ultimately may cause slow microcrack repair and a more brittle bone^24^. Although both bisphosphonates and denosumab reduce the osteoclast activity, their action mechanism is different. Bisphosphonates binds to the bone mineral, preventing the inhibitory effect of mature osteoclasts, while denosumab precludes the binding of RANK-L to its receptor RANK^25^.

The discovery of the RANK/RANK-L/OPG pathway has been an important progress in the understanding of bone remodeling^26–28^. RANK is a protein secreted by the osteoblasts that acts as a receptor at the membrane of precursor osteoclasts^27^, with an important effect in the formation, function, and survival of osteoclasts. Binding of RANK to its ligand (RANK-L) causes the differentiation of precursor osteoclasts to mature osteoclasts^28^, as well as the biochemical signalling between osteoblasts and osteoclasts, controlling bone remodeling. OPG is a decoy receptor for RANK-L^26^ with a higher affinity than RANK. When OPG attaches to the receptor sites in the precursor osteoclast membrane, it precludes RANK/RANK-L binding, reducing bone resorption^27^. In addition to this main pathway, other growth factors, cytokines, and hormones, such as ${TGF_{\beta }}$ and PTH, are also involved in bone homeostasis^27^. A complete understanding of this RANK/RANK-L/OPG pathway and its interaction with the mechanical strain and with external drugs such as those mentioned would help to identifying the optimal dose for patients with bone disorders.

Peter et al.^29,30^ analyzed the effect of antiresorptive drugs on the bone remodeling process in patients with osteoporosis utilizing a finite element model around a hip implant after application of alendronate. They used a phenomenological bone remodeling model to investigate the effect of such drug on the osteoclast activity trying to establish a relation between the drug dose and the parameters of the resorption part of the density rate-stimulus curve. Hambli et al.^31^ investigated the denosumab effect on bone remodeling, considering a couple PK/PD and FE model. Although their work gave rise to good predictions on the mean bone mineral density, they did not consider the effect of different loads, nor different doses on different bone types. Also, their damage model did not consider damage repair. Finally, the effect on long-term mineralization and the damage increase induced by the higher mineralization-induced brittleness were not considered either. Martinez et al.^32^ studied the effect of denosumab on the bone mineral density, but without taking into account the damage effect, neither different types of bones under various loads.

Therefore, the development of a pharmacokinetic-dynamic (PK/PD) model for bone remodeling that also takes into account the mechano-chemical coupling can consequently help in predicting the bone evolution and behavior after implantation in patients with osteoporosis and in optimizing the treatment with different types of drugs. In this work, we investigate the effect of different doses of drugs on bone remodeling with the help of the PK/PD model provided by Marathe et al.^8,33^. This model is complemented here with a sub-model that couples the mechanical signal with the RANK/RANK-L/OPG pathway^34^. Finally, the resulting mechano-PK/PD model is used to analyze the evolution of bone in normal and osteoporotic mandibles after dental implantation with different drug dosages.

## Results

**Figure 1:**
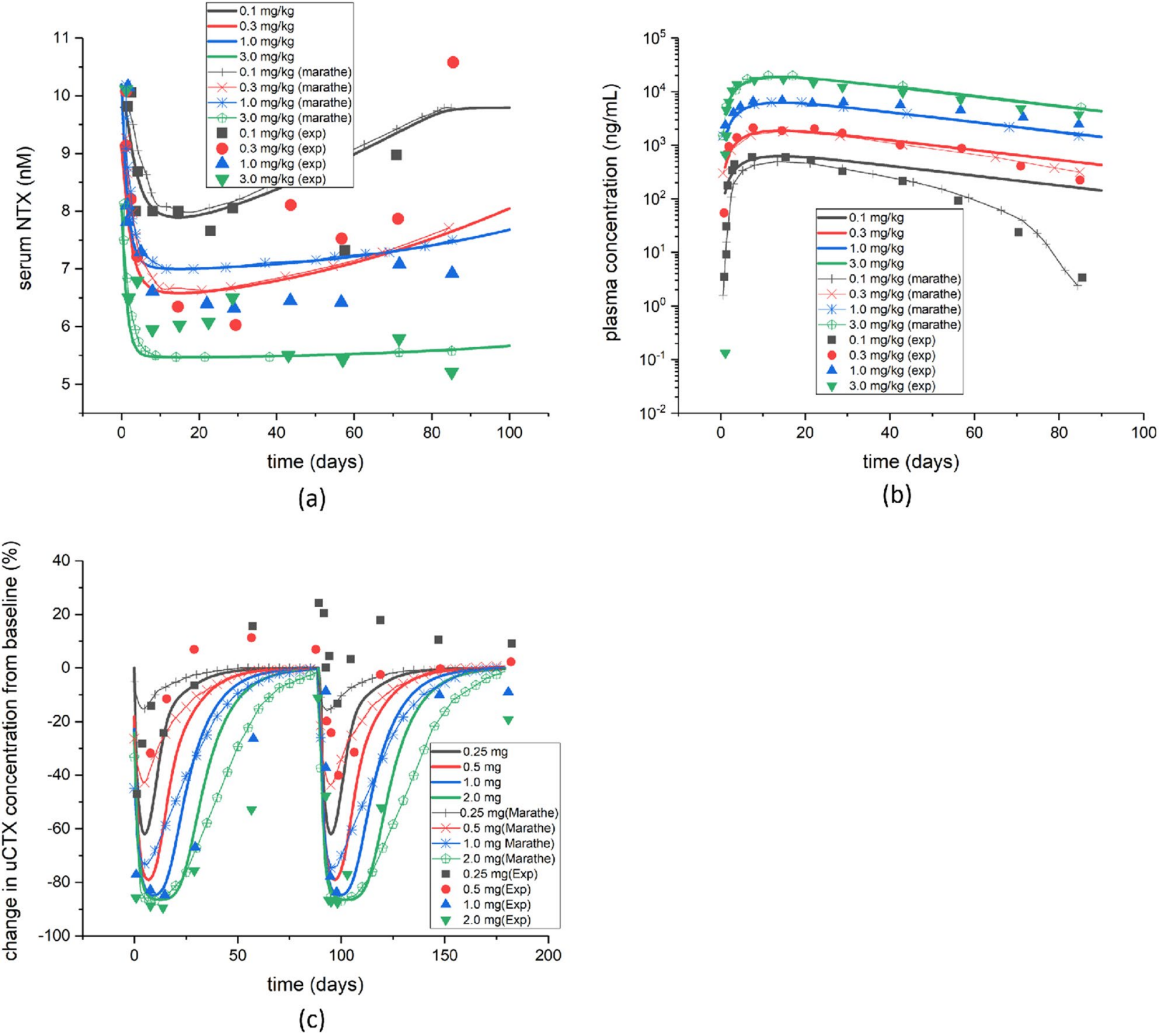
(**a**) Evolution of the serum NTX concentration after administration of a single dose of denosumab and comparison with Marathe’s work^33^ and with experiments^36^; (**b**) evolution of the plasma concentration after administration of a single dose of denosumab and comparison with Marathe’s work^33^ and with experiments^36^; (**c**) changes in the concentration of urine CTX from the baseline after administration of a single dose of Ibandronate and comparison with Marathe’s work^8^ and with experiments^35^.

First of all, we tried to validate the biochemical model described above. With such purpose, we calculated the evolution in time of two biomarkers for bone turnover, Serum N-terminal telopeptide (sNTX), after application of different doses of denosumab, and urine C-terminal telopeptide (uCTX) after application of different doses of Ibandronate. Figure 1a,b show the evolution during 90 days of sNTX and plasma concentrations after administration of a single dose of denosumab. In the first days, a significant decrease in sNTX was observed for any dosage. This reduction is slowly recovered from 55 to 95$\%$ of the initial baseline, in 80 days, depending on the dose. That initial decrease is higher for higher doses, although the difference between doses of 0.3 and 1 mg/kg is small. On the contrary, the plasma concentration increases by orders of magnitude in the first days after drug administration, with subsequent recovery towards the initial baseline. Figure 1c shows the evolution of uCTX concentration after intravenous administration of Ibandronate for 180 days and an interval between successive doses of 90 days. The concentration of uCTX shows a strong reduction in the first days after drug administration, up to values of 80$\%$ of reduction for a dose of 2 mg of Ibandronate. Then the concentration starts to rise towards its initial baseline, which is reobtained at about 90 days after drug administration. All these results are in good agreement with those presented in other studies^8,33,35,36^.

**Figure 2:**
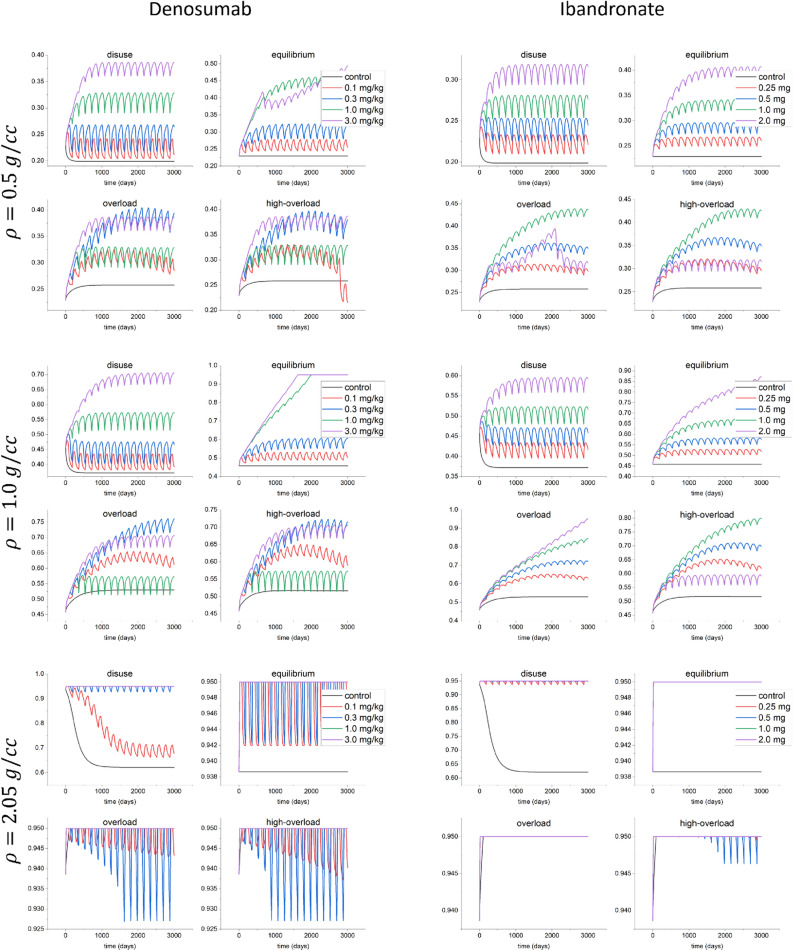
Evolution of the bone volume fraction (Eq. (2)) for the control case without drugs and for different doses of denosumab (0.1, 0.3, 1.0 and 3.0 mg/kg) and Ibandronate (0.25, 0.5, 1.0 and 2.0 mg) when applying different stimuli of disuse ($\xi =0$), equilibrium ($\xi =\xi ^*$), overload ($\xi =5\xi ^*$) and high-overload ($\xi =7\xi ^*$) for different bone types ($\rho =0.5, 1.0$ and $2.05 \,\hbox {g}/\hbox {cm}^3$).

The evolution of the bone volume fraction (Eq. (2)) for different bone types (osteoporotic, $\rho =0.5 \, \hbox {g}/\hbox {cm}^3$, trabecular, $1.0\,\hbox {g}/\hbox {cm}^3$, and cortical, $2.05 \,\hbox {g}/\hbox {cm}^3$) under different mechanical stimuli of disuse ($\xi =0$), equilibrium ($\xi =\xi ^*$), overload ($\xi =5\xi ^*$) and high-overload ($\xi =7\xi ^*$), with $\xi ^*$ denoting the reference stimulus (Eq. (7)) are depicted in Fig. 2. The initial values are obtained by solving the stationary state of Eq. (1), i.e without considering the drug effect and under equilibrium stimulus ($\xi =\xi ^*$). When increasing the drug dose, the volume fraction increases for all types of bone. For $\rho =1.0\,\hbox {g}/\hbox {cm}^3,$ the maximum increase of volume fraction in the equilibrium condition with respect to the control case for 0.1, 0.3, 1.0 and 3.0 mg/kg of denosumab was about 16%, 32%, 53% and 107% respectively, while for 0.25, 0.5, 1.0 and 2.0 mg of Ibandronate these increases were 15%, 27%, 46% and 90% respectively.

**Figure 3:**
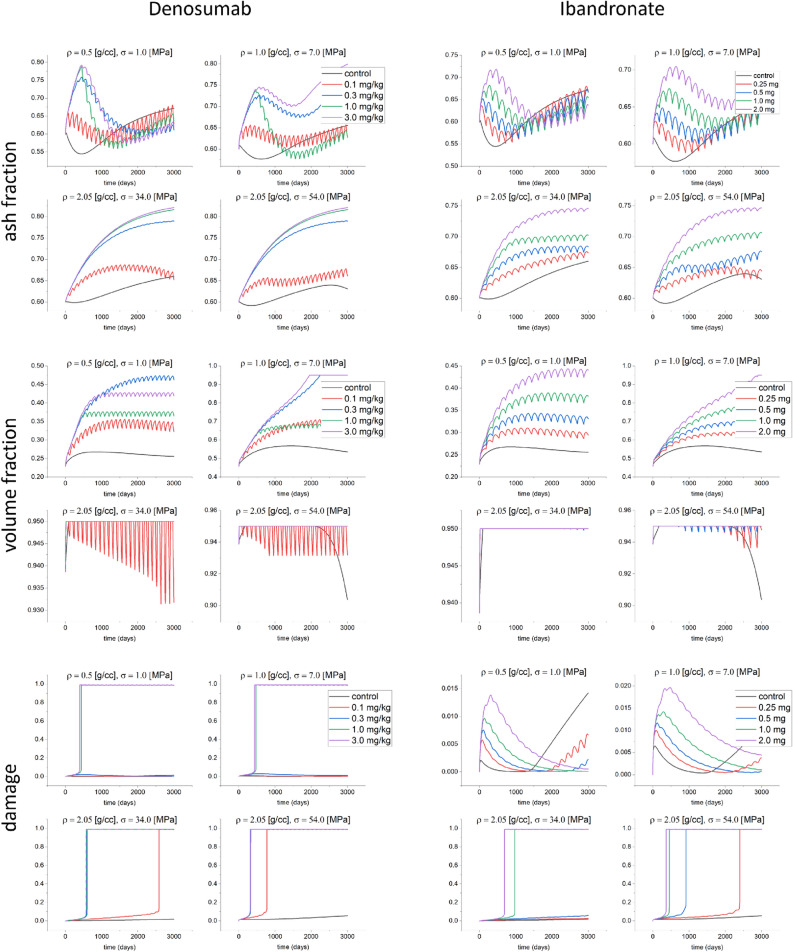
Evolution of the ash fraction, volume fraction and damage for the control case, and different doses of denosumab (0.1, 0.3, 3.0 mg/kg) and Ibandronate (0.25, 0.5, 1.0 and 2.0 mg) when applying constant stress of 1.0 MPa for an initial density of $\rho = 0.5 \,\hbox {g}/\hbox {cm}^3$, 7.0 MPa for an initial density of $\rho = 1.0 \,\hbox {g}/\hbox {cm}^3$ and 34 and 54 MPa for an initial density of $\rho = 2.05\, \hbox {g}/\hbox {cm}^3$.

The evolutions of ash fraction, bone volume fraction and damage are shown in Fig. 3 when the bone is subjected to constant stress values of $\sigma =1.0 \, \hbox {MPa}$ for $\rho =0.5\,\hbox {g}/\hbox {cm}^3$, $\sigma =7.0 \ \hbox {MPa}$ for $\rho =1.0\,\hbox {cm}^3$ and $\sigma =34.0$ and $54 \ \hbox {MPa}$ for $\rho =2.05\,\hbox {g}/\hbox {cm}^3$, that correspond to similar stimuli, and after administration of different doses of denosumab and Ibandronate. As shown in^34^ and in Fig. 3, the ash fraction decreases during the first stage of remodeling, because of the activity of osteoclasts tends to increase. When applying one of those two drugs, the ash fraction increases for all types of bones. In the cases of osteoporotic bone under $\sigma =1.0 \ \hbox {MPa}$, and trabecular bone, the ash fraction first increases, and then decreases for doses of 1.0 and 3.0 mg/kg of denosumab. After this period, the ash fraction begins to increase again. The bone volume fraction increases for all types of bones, as shown in Fig. 3. This same figure also shows that the bone volume fraction tends to decrease with the stress level in trabecular and osteoporotic bones. The same trend can be observed when prescribing bisphosphonates. The bone volume fraction in osteoporotic bone increases in that latter case up to the maximum density allowed. Also, the increase rate for the bone volume fraction is higher when using denosumab than when applying bisphosphonates (Fig. 3). Finally, the damage level in cortical bone ($\rho =2.05\,\hbox {g}/\hbox {cm}^3$) shows a greater increase than for the two other bone types. Using denosumab with doses of 0.1 and 0.3 mg/kg does not increase the damage level in osteoporotic and trabecular bones, while, on the contrary, for cortical bone, for those doses, the damage level increases. In the case of bisphosphonates, doses of 0.25 and 0.5 mg do not increase the damage level for osteoporotic bone. The change in ash fraction for $\rho =1.0\,\hbox {g}/\hbox {cm}^3$ subjected to $\sigma =7.0 \ \hbox {MPa}$ for for 0.25, 0.5, 1.0 and 2.0 mg of denosumab with respect to the control case at time t=3000 days was about – 2%, 14%, – 4% and 20% respectively, while these changes for 0.25, 0.5, 1.0 and 2.0 mg of Ibandronate were about – 0.7%, – 0.9%, – 1.5% and 5% respectively.

**Figure 4:**
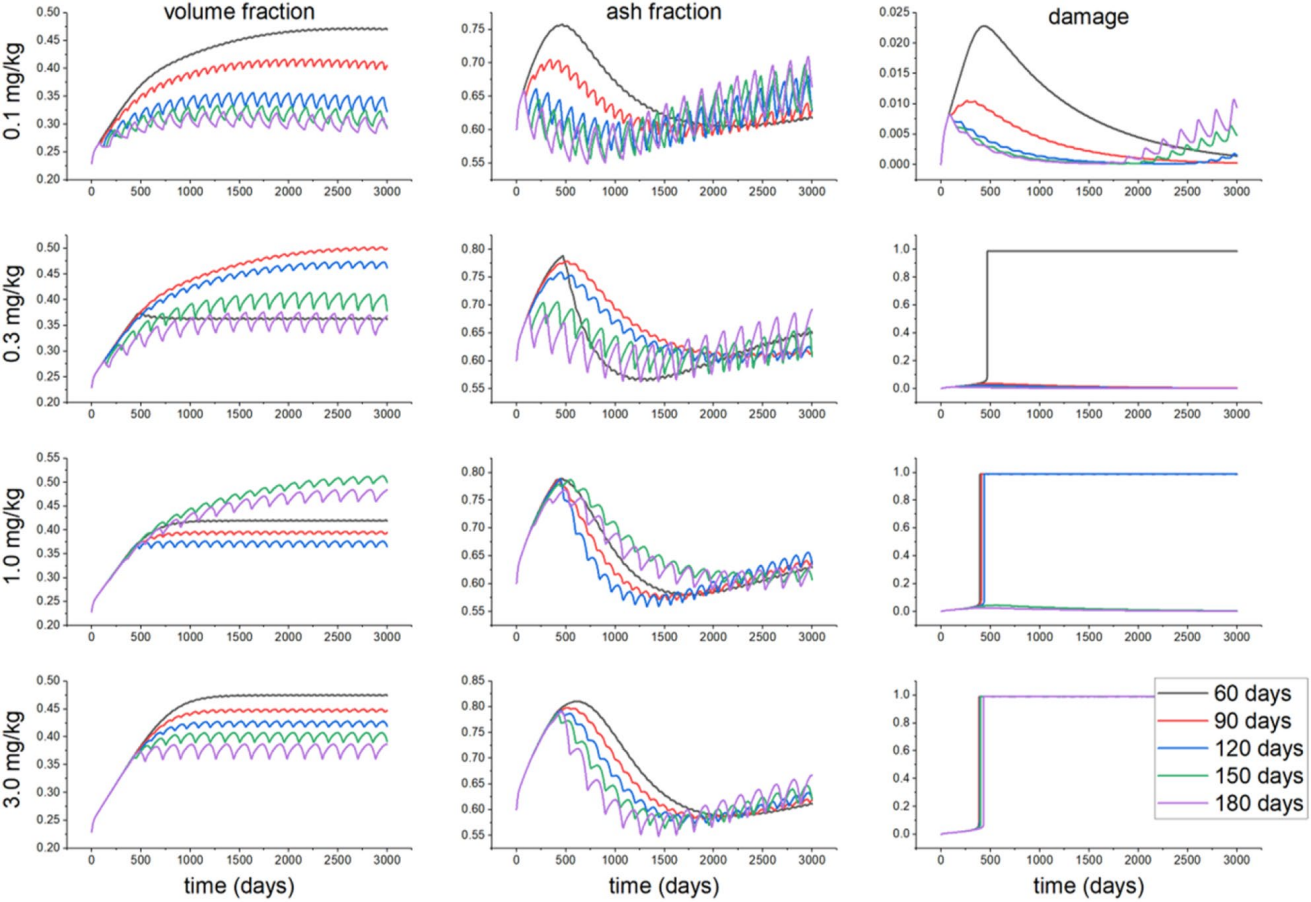
Evolution of the volume fraction, ash fraction and damage for different doses of denosumab (0.1, 0.3, 1.0 and 3.0 mg/kg) when applying a stress of 1.0 MPa for a bone initial density of $\rho = 0.5\, \hbox {g}/\hbox {cm}^3$, and different dosage time intervals of 60, 90, 120, 150 and 180 days.

**Figure 5:**
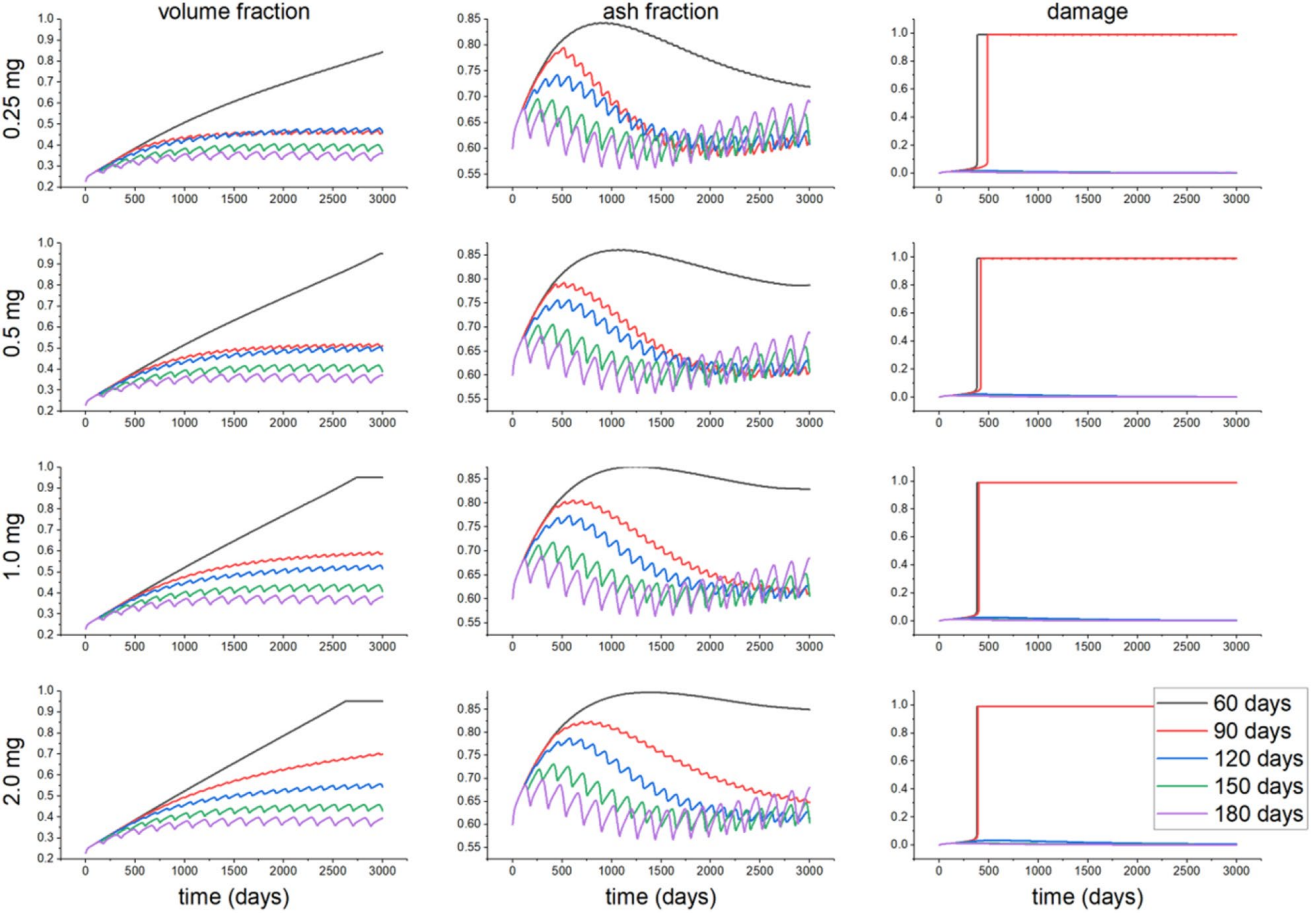
Evolution of the volume fraction, ash fraction and damage for different doses of Ibandronate (0.25, 0.5, 1.0 and 2.0 mg) when applying a stress of 1.0 MPa for a bone initial density of $\rho = 0.5\, \hbox {g}/\hbox {cm}^3$, and different dosage time intervals of 60, 90, 120, 150 and 180 days.

The evolutions of bone volume fraction, ash fraction, and damage for different doses of drugs and different dosage intervals for the osteoporotic bone, in particular, are depicted in Figs. 4 and 5. For a denosumab dose 0.1 mg/kg, increasing the dosage interval decreases the bone volume and ash fractions. In contrast, for a dose of 0.3 mg/kg and a time interval of 60 days, the damage reaches the maximum level allowed. After reaching that maximum damage, the ash fraction shows a higher reduction rate than for other doses. Simultaneously, the volume fraction for this dose is slightly reduced and then reaches a new steady value after the maximum damage is reached. For other time intervals and the same dose, the bone volume fraction increases with time, depending on the time interval, since damage does not reach its maximum level. In contrast, the ash fraction decreases when increasing the time interval. Increasing the drug dose increases damage, while shorter time intervals drive to a faster increase in damage up to its maximum level. Moving now to Ibandronate, a dose of 0.25 mg increases the damage up to its maximum value at time intervals of 60 and 90 days. The ash and bone volume fractions, unlike denosumab, do not stop increasing after reaching maximum damage. The same result is observed for other doses and dosage time intervals. It is possible to observe faster increases in the ash and bone volume fractions when decreasing the dosage time interval for all doses. Reductions in volume fraction for $\rho = 0.5\, \hbox {g}/\hbox {cm}^3$ after 3000 days from the case of 60 days for 90, 120, 150 and 180 days of administration of 0.1 mg/kg of denosumab were about 13%, 31%, 37% and 37% respectively, while for Ibandronate these values were 45%, 45%, 56% and 57% respectively.

**Figure 6:**
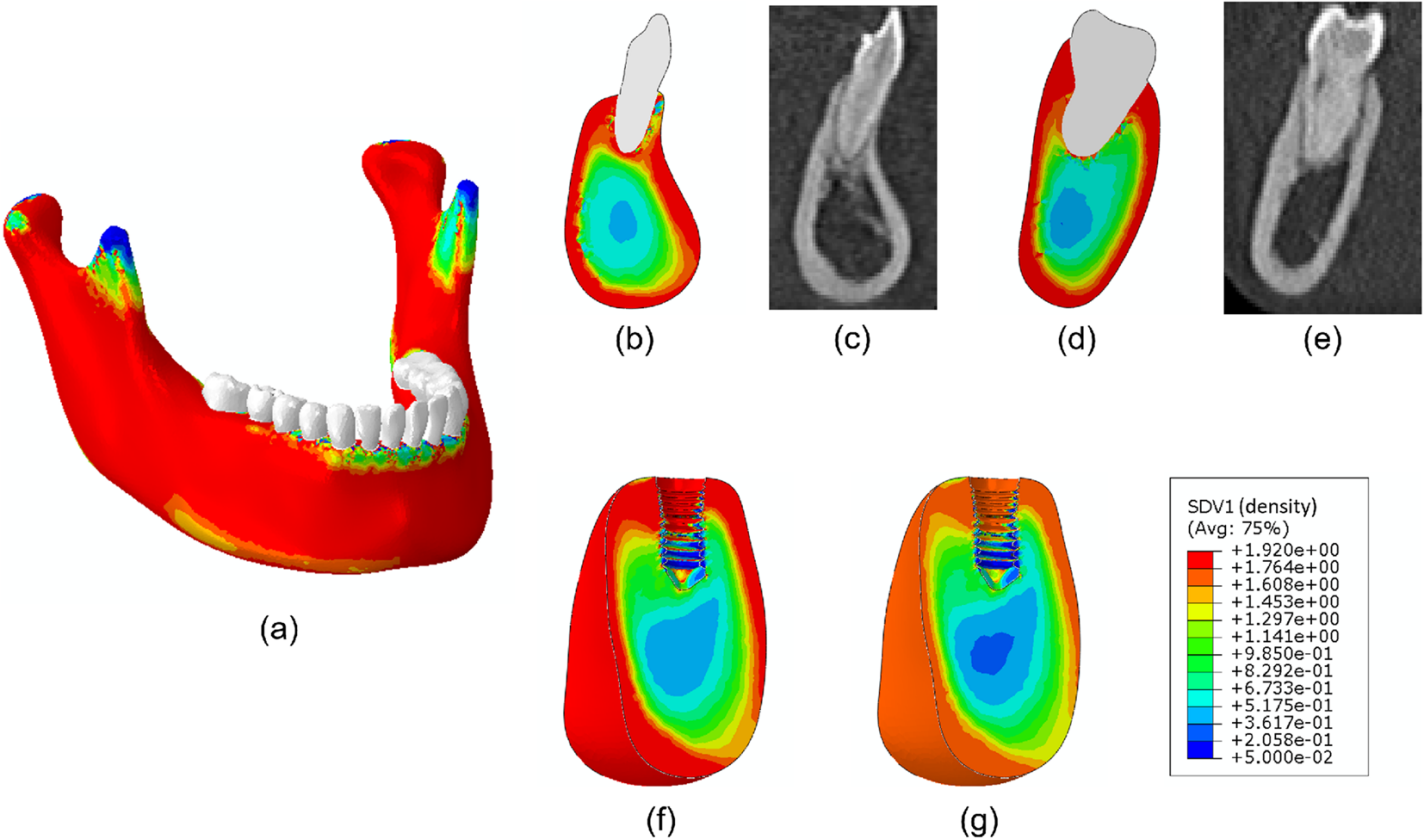
Bone density distribution ($\hbox {g}/\hbox {cm}^3$) in the mandible and several cross-sections. (**a**) Mandible, (**b**) cross section containing the incisor, (**c**) CT image of the corresponding incisor cross section, (**d**) cross section of second right molar, (**e**) CT image of the corresponding second right molar section, (**f**) lingual-labial cut view of the isolated model and (**g**) density of the osteoporotic state in the same cut view.

Figure 6 shows the final density distribution in the whole mandible after application of the phenomenological bone remodeling model with additional views of several cross-sections to compare such results with corresponding CT images. Figure 6f shows a cut view of the density distribution of the reduced computational model, while Fig. 6g depicts the assumed density distribution after applying the density reduction due to osteoporosis.

**Figure 7:**
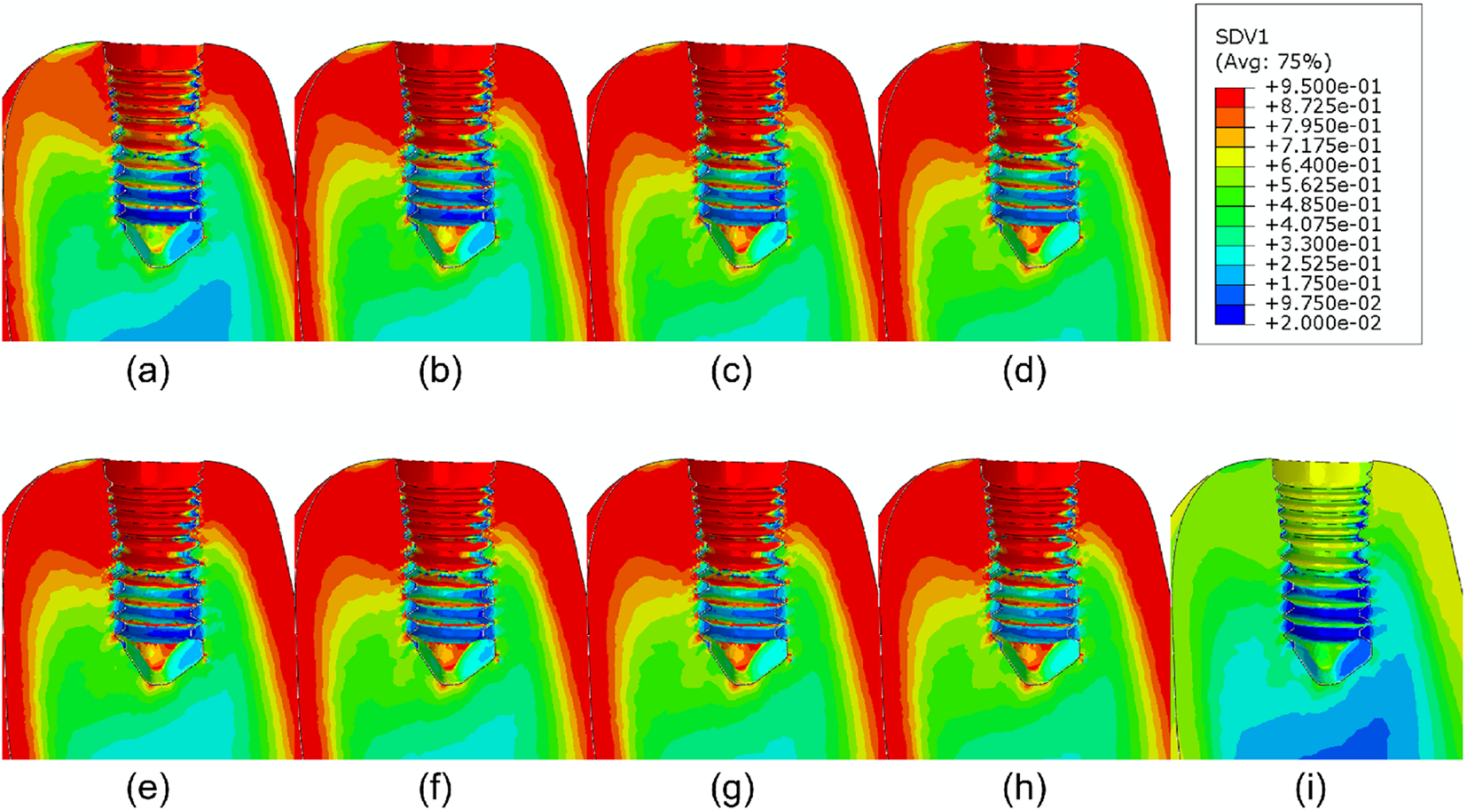
Bone volume fraction distribution after 540 days of simulation for different doses of (**a**) 0.1, (**b**) 0.3, (**c**) 1.0, (**d**) 3.0 mg/kg of denosumab and doses of (**e**) 0.25, (**f**) 0.5, (**g**) 1.0, (**h**) 2.0 mg of bisphosphonates and (**i**) control implant.

**Figure 8:**
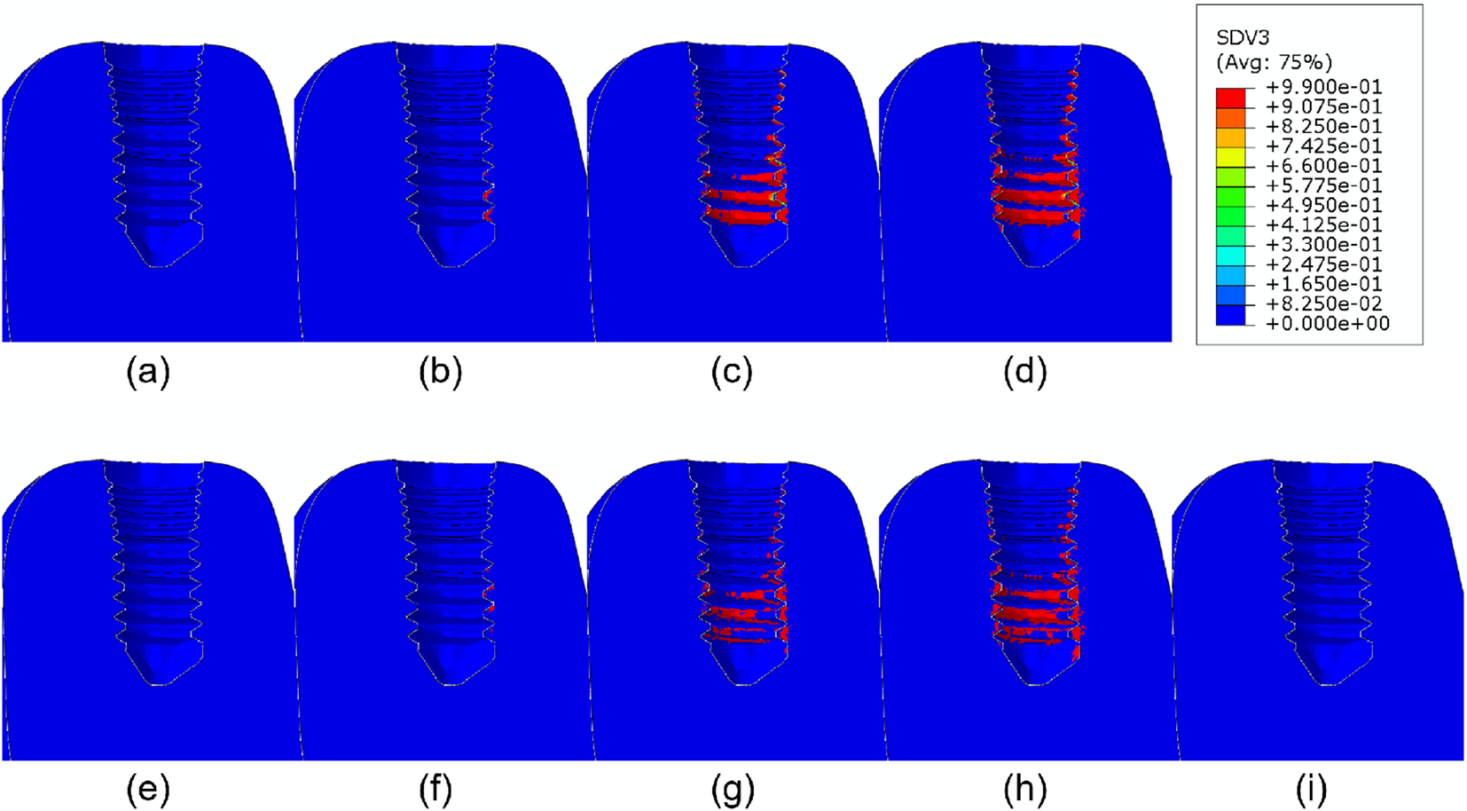
Damage distribution after 540 days of simulation for different doses of (**a**) 0.1, (**b**) 0.3, (**c**) 1.0, (**d**) 3.0 mg/kg of denosumab and doses of (**e**) 0.25, (**f**) 0.5, (**g**) 1.0, (**h**) 2.0 mg of bisphosphonates and (**i**) control implant.

**Figure 9:**
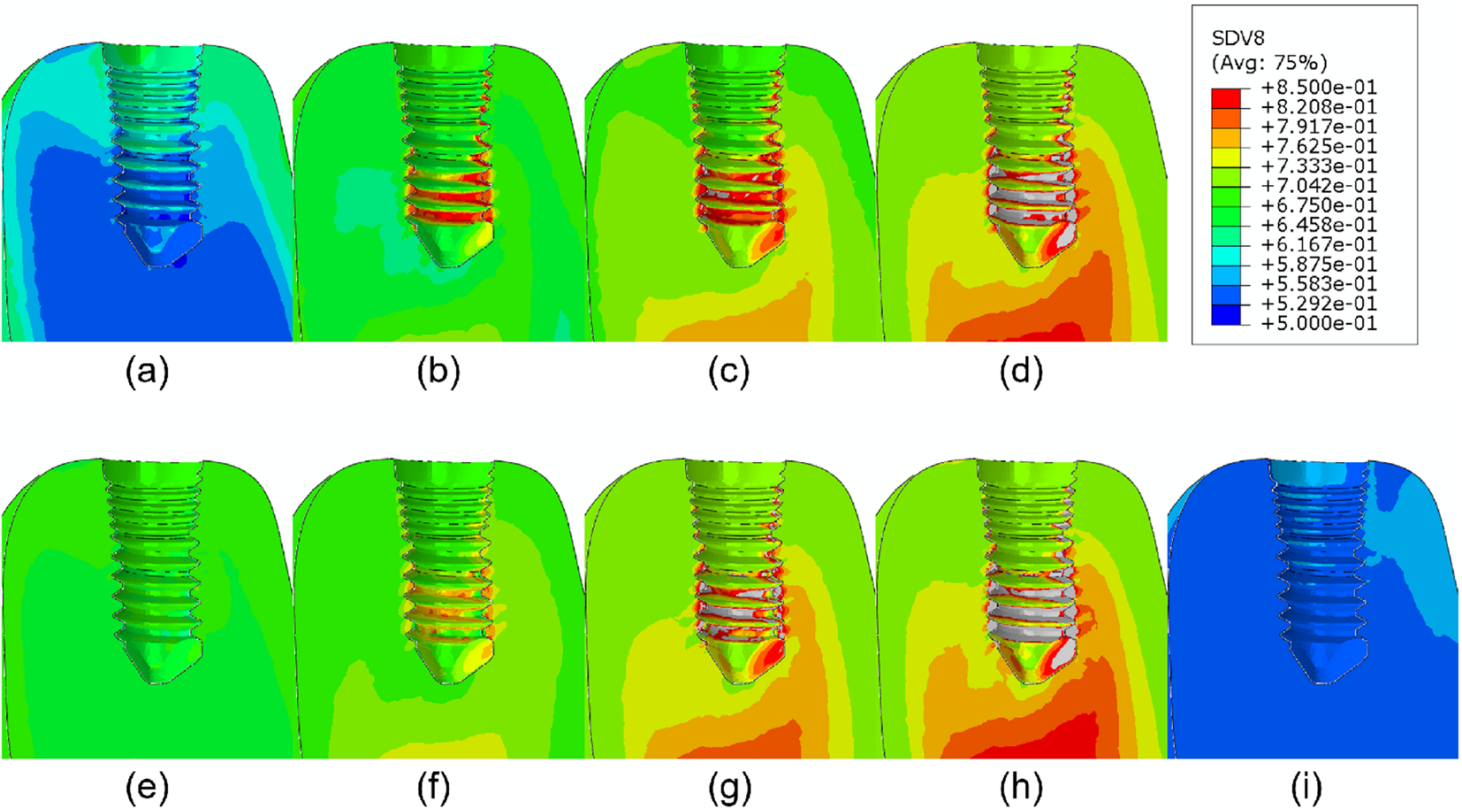
Ash fraction distribution after 540 days of simulation for different doses of (**a**) 0.1, (**b**) 0.3, (**c**) 1.0, (**d**) 3.0 mg/kg for denosumab and doses of (**e**) 0.25, (**f**) 0.5, (**g**) 1.0, (**h**) 2.0 mg for Ibandronate and (**i**) control implant.

**Figure 10:**
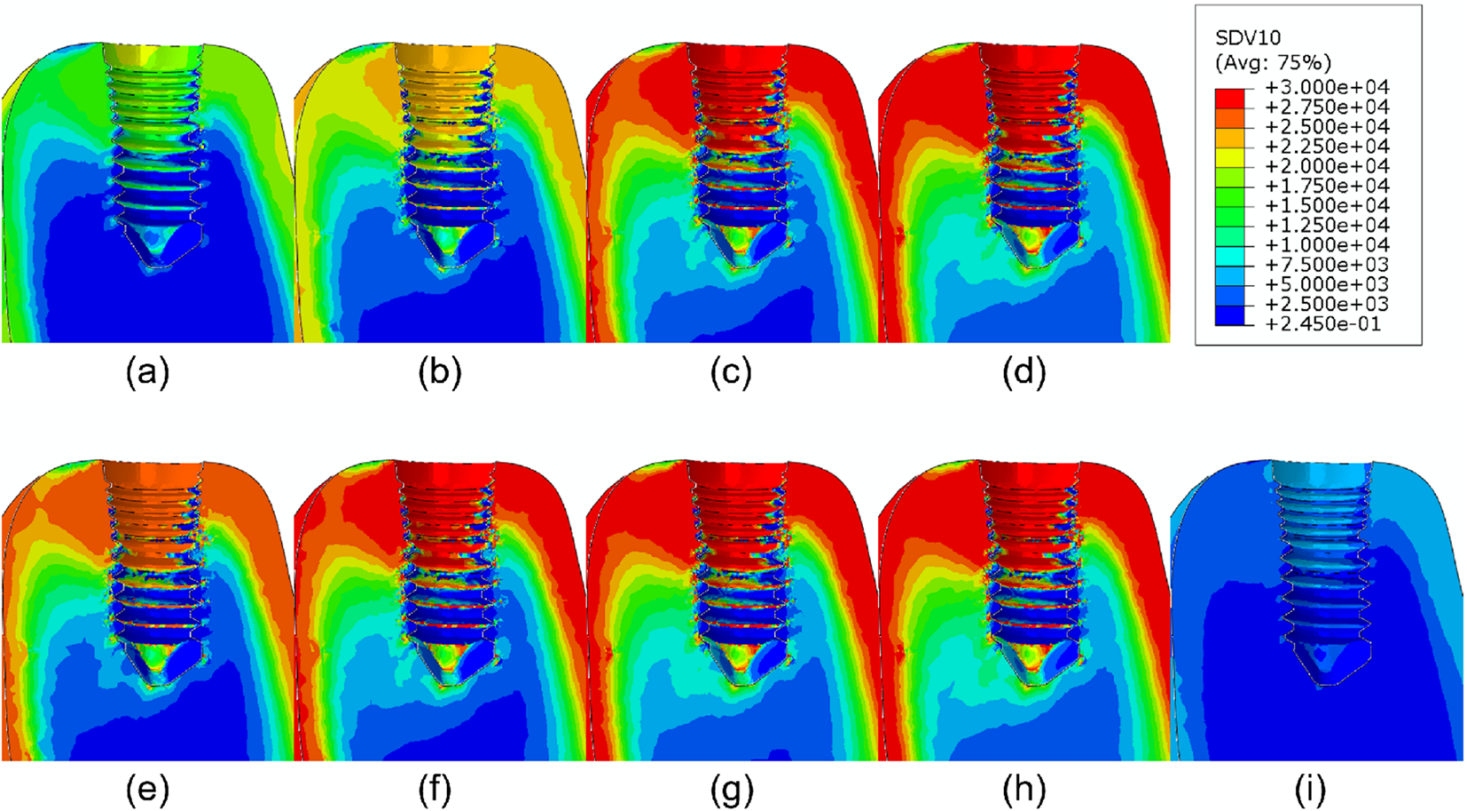
Elastic modulus distribution after 540 days of simulation for different doses of (**a**) 0.1, (**b**) 0.3, (**c**) 1.0, (**d**) 3.0 mg/kg of denosumab and doses of (**e**) 0.25, (**f**) 0.5, (**g**) 1.0, (**h**) 2.0 mg of bisphosphonates and (**i**) control implant.

Finally, the application of the coupled PK/PD and remodeling models here described to the mandible model after dental implantation, physiological mastication loads, and administration of different drug doses drives to the results shown in Figs. 7, 8, 9 and 10. The bone volume fraction (Fig. 7), damage level (Fig. 8) and ash fraction (Fig. 9) are compared with the base case with the application of no drug. For the two drugs analyzed here, any dose increases the bone volume and ash fractions and corresponding density. Doses of 1.0 and 3.0 mg/kg of denosumab and 1.0 and 2.0 mg of bisphosphonate produce higher damage. As consequence, the elastic modulus decreases in those regions (Fig. 10). The ash fraction (Fig. 9), like damage, increases when increasing the drug dose, especially in the trabecular bone around the implant threads.

## Discussion

One of the main objectives of this study was to investigate the effect of different doses of antiresorptive drugs on bone behavior. Antiresorptive drugs are used to treat diseases such as osteoporosis in which the balance between the activity of osteoclasts and osteoblasts is disturbed^37^. Denosumab and Ibandronate are antiresorptive osteoclast-targeting drugs used in the treatment of osteoporosis^25,38^. These drugs affect the bone remodeling process by different action mechanisms. Denosumab binds to RANK-L, reducing the binding between RANK and RANK-L, thereby the concentration of active osteoclasts on the bone surface. Bisphosphonates as Ibandronate, on the contrary, interfere with the osteoclast activity by binding to the bone mineral surface^25^. As a consequence, these drugs reduce the resorption stage in bone remodeling, increasing, therefore, the bone volume fraction and density, and, with that, improving the long-term bone quality in osteoporotic patients. Besides, the mineral content of bone increases, which causes bone to become more brittle and damaged. This may provoke local fractures despite the higher stiffness and strength of the treated bone.

Therefore, this ambivalent effect of antiresorptive drugs makes it difficult to predict their net effect on osteoporotic bone. This process is especially complex when treating with such drugs after implantation since in those case, it is not only the effect of drugs but also the critical change in the mechanical conditions of the surrounding bone which contributes to modifying the long-term bone internal microstructure. Implantation in patients with osteoporosis is, therefore, challenging^39^ and its clinical treatment utilizing these drugs may negatively affect the success of the implant with increasing osteonecrosis^24,38^.

This happens, for example, after dental implantation, a practice that has increased in recent years in the elderly, who have an increased risk of osteoporosis in the mandible bone, which justifies why this problem has attracted the interest of several authors^14,15,40,41^. In particular, mathematical models are useful in analyzing these complex problems. In principle, an ideal mathematical model of bone remodeling should take into account the different bone cells involved and their main activities such as proliferation, differentiation, migration, death, biochemical signals production, changes in their expression due to biochemical or mechanical signals, and tissue resorption or production. Also, it should consider the diffusion, decay and production of growth factors, hormones, proteins, and other biochemical substances that control the cell behavior. Finally, the particular mechanical microenvironment and its interactions with the chemical reactions and cell protein expression should be taken into account. To implement all these processes requires very complex mechano-chemo-biological models with several coupled mechanisms not yet fully understood, a lot of parameters, many times unmeasured, and a difficult validation due to lack of experimental results in a sufficient number and variety of situations. Even with these limitations, mathematical modeling is a powerful tool for studying complex biological systems since they allow us to find out important trends, and to quantify, to a certain extent, the relationships between causes and effects, to test theoretical hypotheses, quantify the effects of the different parameters individually on the behavior of the biological system and to do virtual experiments in “what if” situations^42^.

In this paper, we present a combination of a PK/PD model and a fully-coupled chemo-mechano-biological bone remodeling approach that incorporates the stimulus effect on the signaling pathway between osteoclasts and osteoblasts. The effect of damage on the signaling pathway and the local material properties have also been considered. Finally, the mineralization level is monitored along the whole bone lifetime. We have proven that these types of models can be used for predicting the bone behavior in the mandible after dental implantation when using antiresorptive drugs for improving the long-term quality of osteoporotic bone. In order to study the applicability of this model, the long-term effects of different doses of denosumab (0.1, 0.3, 1.0 and 3.0 mg/kg) and Ibandronate (0.25, 0.5, 1.0 and 2.0 mg) on the mandibular bone surrounding a dental implant were studied.

We first examined the effect of different drugs and of the mechanical stimulus on the bone behavior. Denosumab does not bind to the bone mineral surface, unlike Ibandronate, so Ibandronate effects last longer after stopping its administration^24,43,44^. Considering this fact and comparing the increase in bone volume fraction after administration of these two drugs, we can speculate that the increase in volume fraction and in mineral content in bone induced by Ibandronate will be higher than that of denosumab. As commented, the drugs here analyzed inhibit the activity of osteoclasts, thereby reducing bone resorption and therefore, increasing the bone volume fraction and the mineralization. On the other hand, due to the effect of calcium, the bone becomes more brittle, and damage increases. By comparing Figs. 4 and 5, at low doses of 0.1 mg/kg for denosumab and 0.25 mg for Ibandronate, we found a more significant increase in the bone volume fraction when applying Ibandronate, while, contrarily, this drug produces higher increases in bone ash fraction and damage than denosumab, leading to a more brittle bone. Also, these drugs increase the ash fraction initially (Fig. 3), while the mineralized portion of the bone is also initially reduced. This stage is then followed by the filling of the resorbed bone by the osteoblasts, forming new bone with the corresponding next mineralization. Increasing the drug dose in any of two cases considered also increases the bone volume fraction and damage for all types of bone. For cortical bone, for instance, which does not need drug treatment, even low drug doses cause bone to become highly brittle, reaching the maximum level of damage faster. In this bone, no decrease in the mineralized fraction was detected after drug treatment, so microdamage progresses in time, and a stress fracture may occur.

The stress level also influences this behavior by promoting or delaying the damage rate. For trabecular or osteoporotic bone, low drug doses increase the bone volume fraction without substantial damage increase. This is more evident in osteoporotic bone. Consequently, drug application is beneficial in low-density bone, although this treatment always increases brittleness, which may compromise the success of dental implantation. This subtle control between these two opposite effects is essential when defining the treatment protocol for a particular patient and the main reason for using and improving this type of mathematical models and “in silico” experiments.

After this first test of the model, when we got similar results to other authors with comparable qualitative clinical conclusions^20–23^, we studied their effect on the bone surrounding a realistic dental implant using a 3-dimensional finite element model for an osteoporotic mandible after implantation and treatment with different drug doses. Qualitative comparison of the results with clinical studies in patients with osteoporosis who received bisphosphonate or denosumab agrees with the detected bone mineral content and bone mass increase after anti-resorptive treatment^45,46^.

Bone brittleness is not only linked to low bone mass but also to the increase in the mineral content of the tissue or to the accumulation of bone microdamage^47^. This latter could be due to bone resorption and the associated bone strength or to microcrack blockage of the bone remodeling activity, reducing microcracks repair^48^. Another possibility is associated with the use of bisphosphonates or denosumab, since these drugs reduce the activity of osteoclasts, thereby reducing the bone remodeling rate, thus slowing down microdamage repair. As shown in our results, depending on the stress level, the bone type, and the drug dose, the damage level could highly increase when using these types of drugs. For example, in Fig. 3 for osteoporotic bone $\rho = 0.5\, \hbox {g}/\hbox {cm}^3$, after applying constant stress of $\sigma =1.0 \ \hbox {MPa}$ the amount of volume fraction increased along the whole process (43% for 0.3 mg/kg of denosumab and 30% for 0.5 mg of Ibandronate), but the increase in ash fraction in the early days leads to a maximum in the damage level. A similar effect is seen in Figs. 7 and 8. The first one shows an increase in the bone volume fraction in the patient with osteoporosis for higher doses of antiresorptive drugs. This increase is also seen in the vicinity of the implant threads. On the other hand, the second figure shows that microdamage also increases, especially in the threads neighborhood, which confirms the findings of other studies^47,49,50^.

Despite the remarkable potential of these models and the qualitatively accurate results produced, like any other mathematical models in systems biology, it still has important limitations that should be addressed in future studies. Among them, we can mention:

1. As mentioned in our previous study^34^, simplifications such as considering only the RANK/RANK-L/OPG biochemical pathway, or in the reaction rate assumed for the receptor–ligand reactions, or discarding the effect of osteocytes in the mechanosensing process, should be progressively overcome.
2. Due to the lack of knowledge about the effect of race, sex, age, etc, onto the biological parameters, those used in this study are taken from literature, so it is not possible yet to apply the model to patient-specific studies without additional experimental determinations.
3. The pharmacokinetics determines the dosage of the drug in the intended compartment. Therefore, developing a pharmaco-kinetic model with an adequate number of compartments and experimental parameters is essential for determining an accurate value for the concentration of drug reaching bone to better predict the bone behavior. Also, the parameters used in pharmacokinetic models should vary for different individuals, which has not been considered here.
4. Although the mineral part of the bone has a prevailing effect on the bone mechanical properties^51^, the effect of drugs on the organic component of bone was not considered, which may be important to assess bone fracture resistance, especially under tension^52^.
5. Different loading conditions have different effects on bone behavior. However, due to the lack of sufficient information, and considering that muscle forces vary for different individuals and are also modified after implantation, the loads applied in our finite element study were based on previous generic studies.

We have presented here a PK/PD model coupled with a mechano-chemo-biological bone remodeling model, that has been implemented in a standard commercial FE code to analyze the effect of two widely used drugs as denosumab and Ibandronate onto the evolution of the mandible bone after dental implantation. Using antiresorptive agents such as denosumab or bisphosphonates alter the evolution of the density and mechanical properties of bone by interfering in the bone remodeling mechanism, reducing the osteoclast activity, which may consequently increase bone brittleness by augmenting the mineral content and microdamage. This effect is corroborated by our results that show that using any of those drugs in osteoporotic patients increases the bone volume fraction, although, in parallel, it also increases bone brittleness, a well-known side effect of these treatments. Using patient-specific geometries and initial density values, this model may provide a good perspective to clinicians about these two contradictory effects of those drugs for the treatment of osteoporosis and to compare with longitudinal clinical results with different doses.

The model developed here is capable of capturing the main biological and chemical variables associated with the bone remodeling process in patients receiving drugs for osteoporosis treatment. Despite the limitations described, these models can be used to predict bone behavior, complementing costly, and time consuming clinical experiments, and for optimizing dental implant designs and coatings for osteoporotic patients, as well as patient-specific dosage protocols.

## Methods

The bone remodeling model used here is a chemo-mechano-biological model that couples the effect of mechanical strains and microdamage with the biochemical RANK/RANK-L/OPG pathway, and, with the expression of the different cell phenotypes involved. It also models the tissue resorption-formation process accomplished by synchronized sets of osteoclasts and osteoblasts known as basic multicellular units (BMUs), followed by the tissue mineralization. This model closely follows the one presented in a previous paper of the authors^34^ but adding now the PK/PD submodel that permits the analysis of the effect of drugs. As a result, the whole process and the effect on its main output variables, such as the local bone density and the mineral content, as well as their influence onto the bone mechanical properties, can be analyzed for different mechanical or chemical protocols.

### Bone remodeling model

Regarding the bone remodeling model, only a brief review is done here. For additional details, the reader may consult^34^. That mechano-chemo-biological model is based on the previous work of Lemaire et al.^27^. Three types of cells are considered: responsive osteoblasts, active osteoblasts, and active osteoclasts. The concentration rate (time derivative of the number of cells per unit volume) for each of these three cell populations is written as in Lemaire’s :

1 $$\begin{aligned} \begin{aligned} \frac{dB_r}{dt}=D_{R} {\pi }_{TGF_{\beta }}-\frac{D_{B}}{ {\pi }_{TGF_{\beta }}}B_r \\ \frac{dB_a}{dt}=\frac{D_{B}}{ {\pi }_{TGF_{\beta }}}B_r-k_{B}B_a \\ \frac{dC}{dt}=D_{C} {\pi }_{RANK-L}-D_{A} {\pi }_{TGF_{\beta }}C \end{aligned} \end{aligned}$$

where $B_r$, $B_a$ and *C* identify the concentrations of responding and active osteoblasts and osteoclasts, respectively. ${\pi }_{TGF_{\beta }}$ and ${\pi }_{RANK-L}$ are the fraction of receptor sites occupied by receptor/ligand complex (i.e. $\pi \sim R\bullet L/R$) of the transforming growth factor ($TGF_{\beta }$) and of RANK-L, respectively. $D_{R}$, $D_{B}$ and $D_{C}$ are differentiation rates of osteoblast progenitors to responsive osteoblasts, responsive osteoblasts to active osteoblasts, and osteoclasts precursors to osteoclasts, respectively. $D_{A}$ is the osteoclasts apoptosis rate caused by $TGF_{\beta }$ and, finally, $k_{B}$ is the death rate of active osteoblasts. The values of all these parameters appear in Table 1 with the corresponding references.

**Table 1:** Values and description of the chemical parameters.

	Description	Unit	Value	References
$\beta$	Bond interaction constant	–	${v_b}/v_{b_0}$	–
$C^S$	Value of C (osteoclast population) to get half differentiation flux	pM	5e${}^{-3}$	^27^
$D_A$	Rate of osteoclast apoptosis caused by TGF $\beta$	day${}^{-1}$	0.7	^27^
$d_B$	Differentiation rate of responsive osteoblasts	day${}^{-1}$	0.70	^27^
$D_C$	Differentiation rate of osteoclasts precursors	pM day${}^{-1}$	2.1e${}^{-3}$	^27^
$D_R$	Differentiation rate of osteoblast progenitors	pM day${}^{-1}$	7e${}^{-4}$	^27^
$f_0$	Fixed proportion	–	0.05	^27^
*K*	Fixed concentration of RANK	pM	10	^27^
$k_1$	Rate of OPG-RANK-L binding	pM${}^{-1}$ day${}^{-1}$	10${}^{-2}$	^27^
$k_2$	Rate of OPG-RANK-L unbinding	day${}^{-1}$	10	^27^
$k_3$	Rate of RANK/RANK-L binding	pM${}^{-1}$ day${}^{-1}$	$5.8 \times 10{}^{-4}$	^27^
$k_4$	Rate of RANK/RANK-L unbinding	day${}^{-1}$	$1.7 \times 10{}^{-2}$	^27^
$k_5$	Rate of PTH binding with its receptor	pM${}^{-1}$ day${}^{-1}$	0.02	^27^
$k_6$	Rate of PTH unbinding	day${}^{-1}$	3	^27^
$K^P_L$	Maximum number of RANK-L attached on each cell surface	pmol/pmol cells	$3 \times 10{}^{-6}$	^27^
$k_0$	Rate of OPG removal	day${}^{-1}$	0.35	^27^
$K^P_O$	Minimal rate of production of OPG per cell	pmol day${}^{-1}$/pmol cells	$2 \times 10{}^{5}$	^27^
$k_p$	Rate of PTH removal	day${}^{-1}$	86	^27^
$r_L$	Rate of RANK-L production and elimination	pM day${}^{-1}$	10${}^{3}$	^27^
$S_p$	Rate of synthesis of systemic PTH	pM day${}^{-1}$	250	^27^

After calculating the osteoblast and osteoclast populations, the rate of bone volume fraction can then be calculated as:

2 $$\begin{aligned} \frac{dv_{b}}{dt}=k_{form}B_a-k_{res}C \end{aligned}$$

where $k_{form}$ and $k_{res}$ are the rates of bone formation and resorption per unit cell, respectively, that depend on the bone type and location. The initial value of their ratio $k_{form}/k_{res}$ is assumed to be equal to the initial ratio of the population of osteoclasts and osteoblasts $C_{0}/{B_a}_{0}$, that can be obtained by solving the steady-state expression of Eq. (1) (left-hand side equal to zero) and depend on the initial bone density.

Equations (1) and (2) provide the evolution along time of the distributions of the different cells involved in the process depending on the results of the chemical reactions between receptor and ligands involved in the RANK/RANK-L/OPG pathway that is briefly explained in the following section, and from them, the change in bone volume fraction.

### Chemical reactions involved in bone remodeling

The receptor activator for nuclear kappa-B, RANK (*K*) is a surface-bound molecule that binds to its ligand, RANK-L (*L*), serving as osteoclast activator^28^. Osteoprotegerin, OPG (*O*), a decoy receptor for RANK-L, is another protein expressed by osteoblasts and other tissues like the spleen, bone marrow, heart, liver, and kidney^26^. OPG inhibits RANK/RANK-L binding, so it plays a protective role against bone loss. Among the many systemic hormones that influence bone cell activity, PTH (*P*) is the most important calcium homeostasis regulator and bone remodeling hormone, so it is used as an anabolic agent in the treatment of osteoporosis^27^. The chemical reactions between the receptors and associated ligands are as follows^27^:

3 $$\begin{aligned} \begin{array}{c} \begin{array}{c} p_O \\ \downarrow \end{array} \\ \begin{array}{c} O \\ \downarrow \end{array} \\ d_O \end{array} + \begin{array}{c} \begin{array}{c} p_L \\ \downarrow \end{array} \\ \begin{array}{c} L \\ \downarrow \end{array} \\ d_L \end{array} \ \begin{array}{c} k_1 \\ \rightleftharpoons \\ k_2 \end{array} \ O\bullet L, \begin{array}{c} \begin{array}{c} p_L \\ \downarrow \end{array} \\ \begin{array}{c} L \\ \downarrow \end{array} \\ d_L \end{array} +K\ \begin{array}{c} k_3 \\ \rightleftharpoons \\ k_4 \end{array} \ K\bullet L, \begin{array}{c} \begin{array}{c} p_P \\ \downarrow \end{array} \\ \begin{array}{c} P \\ \downarrow \end{array} \\ d_P \end{array} +P_r\ \begin{array}{c} k_5 \\ \rightleftharpoons \\ k_6 \end{array} \ P_r\bullet P \end{aligned}$$

with $k_i \ (i=1,3,5)$ reaction binding rates, and $k_i \ (i=2,4,6)$ reaction unbinding rates. $p_O$, $p_L$ and $p_P$ are production fluxes and $d_O$, $d_L$ and $d_P$ are destruction fluxes of OPG, RANK-L and PTH respectively. Since the reactions related to chemical bindings in biological systems occur faster than cell population changes, occupancy of the complexes ($\pi _{L}=\frac{K \bullet L}{K}$ and $\pi _{P}=\frac{P_{r}\bullet {P}}{R_{T}^{P}}=\frac{P}{P+P^s}$) as well as the OPG concentration (*O*) have been considered as pseudo-steady during the whole remodeling process. With all this, the OPG concentration and the fractions of RANK-L and PTH can be calculated as follows^27^:

4 $$\begin{aligned} \begin{aligned} {\pi }_{RANK-L}&=\frac{K \bullet L}{K}=\frac{k_3}{k_4}\frac{K^P_L{\pi }_{PTH}B_a}{1+\frac{k_3}{k_4}K+\frac{k_1}{k_2}O}\\ {\pi }_{PTH}&=\frac{P_r \bullet P}{R^P_T}=\frac{P}{P+P^s}\\ O&=\frac{K^P_O}{k_O.{\pi }_P}B_r \end{aligned} \end{aligned}$$

where $P_{r}$ is the number of free receptor of PTH, $R_{T}^{P}$ stands for the number of PTH receptors per cell, $P^s=k_6/k_5$, $K^P_L$ is the maximum occupancy of RANK-L attached to each cell surface, $P=\frac{S_P}{k_P}$ is the PTH concentration, $S_P$ is the synthesis rate of systemic PTH, $K^P_O$ represents the minimal rate production of OPG per cell and $k_O$ is the rate of OPG removal.

From Eqs. (1)–(4) we can calculate the evolution of $B_r$, $B_a$, *C* and $v_b$ without considering the effect of the mechanical environment on bone signaling. The next sections address this coupling between Mechanics and Biochemistry in bone remodeling.

### Role of mechanical signal in chemical reactions

Mechanical strain and microdamage are the most important signals affecting the receptor–ligand binding/unbinding energy fraction. In particular, mechanical forces change the energy barrier of the molecules and disrupt the binding/unbinding of receptors and ligands in chemical reactions, while damage interrupts the communication channels between cells, thus reducing the level of the mechanical signal. Therefore, we hypothesized in a previous paper^34^ that the reaction rates of the receptor and ligand change as follows:

5 $$\begin{aligned} \begin{aligned} k_f&=k_{f_0}{{e}^{\gamma (1-S){{\hat{S}}_v}}} \\ k_r&=k_{r_0}{e}^{-\gamma (1-S){{\hat{S}}_v}} \end{aligned} \end{aligned}$$

where $k_r$ and $k_f$ are the unbinding and binding rates associated to the receptor and ligand interactions, $k_{r_0}$ and $k_{f_0}$ are unbinding and binding rate constants, $\gamma$ is a constant relating the ratio between current and initial bone volume fractions ($\gamma =\frac{v_b}{v_{b_0}}$), *S* the normalized mechanical signal (see below) and ${{\hat{S}}}_v$ is the normalized specific bone surface (${{\hat{S}}}_v=\frac{S_v}{S_{v_{max}}},$ with $S_{v_{max}}=4.17 \hbox {mm}^2/\hbox {mm}^3$ the maximum specific bone surface available)^53^.

The mechanical signal (that is here considered as inhibitory of the action of the cells^54,55^), depends on the mechanical stimulus ($\xi$) and on the damage level (*d*). We assume that the transmission of this signal in bone depends on its microstructure through the local bone volume fraction (i.e. $v_{b}$). In other words, that signal is transmitted easier in cortical than in trabecular bone and much easier than in osteoporotic bone. With all this, and following^55^, we can write:

6 $$\begin{aligned} S=\frac{\xi }{\xi +c}{\left( 1-d\right) }^{a {v_b}} \end{aligned}$$

with *c* and *a* model parameters that, together with $\xi$, *d* and $v_b$ control the value of the inhibitory remodeling signal. The mechanical stimulus depends on an equivalent strain ${\overline{\varepsilon }}$ and the number of loading cycles *N* as in^56^:

7 $$\begin{aligned} \xi ={\left( \sum _i{N_i{{{\overline{\varepsilon }}}_i}^m}\right) }^{{1}/{m}} \end{aligned}$$

with ${\overline{\varepsilon }}=\sqrt{2u/E}$^55,57^, being *u* the strain energy density and *E* the elastic modulus, that correlates with the bone volume fraction and ash density as stated in the Eq. (21) below. Finally, $m=4$ is an experimental constant^56^ and *i* is the number of different load types.

### Drug effect on bone remodeling

Different therapies for bone diseases such as osteoporosis have been developed, influencing the RANK/RANKL/OPG pathway. For example, denosumab is one of such drugs that binds with RANK-L, reducing its binding potential with its receptor RANK^25^, and with that diminishing the differentiation to osteoclasts and their subsequent activation. In our model, this effect is modelled by adding the following additional reaction in Eq. (3)^33^:

8 $$\begin{aligned} \begin{array}{c} \begin{array}{c} p_{L}\\ \downarrow \\ \end{array} \\ \begin{array}{c} L\\ \downarrow \\ \end{array} \\ d_{L}\\ \end{array} +C_{d} \begin{array}{c} k_{on}\\ \rightleftharpoons \\ k_{off}\\ \end{array} L \bullet C_{d} \end{aligned}$$

where $C_{d}$ is the drug concentration in the plasma compartment, and $k_{on}$ and $k_{off}$ are the association and dissociation rate constants of that reaction, respectively. After inclusion of this reaction, ${\pi }_{RANK-L}$ changes in (4) such as^33^:

9 $$\begin{aligned} {\pi }_{RANK-L}=\frac{K \bullet L}{K}=\frac{k_{3}}{k_{4}}\frac{K_{L}^{P} \pi _{P}B_a}{1+\frac{k_{3}}{k_{4}}K+\frac{k_{1}}{k_{2}}O+\frac{k_{on}}{k_{off}}C_{d}} \end{aligned}$$

with $K_{L}^{P}$ the maximum number of RANK-L attached to each cell surface^27^.

Another drug usually used to treat osteoporosis is Ibandronate. Ibandronate, like other bisphosphonates, adheres to the bone mineral, preventing the activity of mature osteoclasts^25^. As a consequence, the osteoclast population (*C*) in Eq. (1) decreases^8^. In our model, we take this into account by adding the following equation:

10 $$\begin{aligned} \frac{dC}{dt}=D_{C} {\pi }_{RANK-L} \left( 1-\frac{I_{max}C_{b}}{IC_{50}+C_{b}} \right) -D_{A} {\pi }_{TGF_{\beta }}C \end{aligned}$$

where $D_A$ and $D_C$ are the osteoclast apoptosis rate caused by $TGF_{\beta }$ and the differentiation rate of osteoclast precursors to osteoclasts, respectively, as defined in Eq. (1), $I_{max}$ is the maximal fractional extent of inhibition, $IC_{50}$ the concentration producing 50$\%$ of maximal inhibition and $C_{b}$ the concentration of bisphosphonate.

To calculate the drug concentrations ($C_d$ and $C_b$), a pharmacokinetic–pharmacodynamic (PK/PD) model has been used^8,33,35,58^. Following^33^, the PK/PD model equations for subcutaneous administration of denosumab may be stated as:

11 $$\begin{aligned} \begin{aligned} \frac{dC_{tot}}{dt}&=k_a Dose \times e^{-k_at}/(V_c/F)-k_{int}C_{tot}-(k_{el}-k_{int})C_d, \ C_{tot}(0)=0 \\ C_d&=\frac{1}{2}(C_{tot}-R{ss}-K_D+\sqrt{(C_{tot}-R_{ss}-K_D)^2+4K_D C_{tot}}) \\ \frac{dNTX}{dt}&=k_{in} \Big (1-\frac{I_{max}C_d}{IC_{50}+C_d}\Big )-k_{out}NTX \end{aligned} \end{aligned}$$

**Figure 11:**
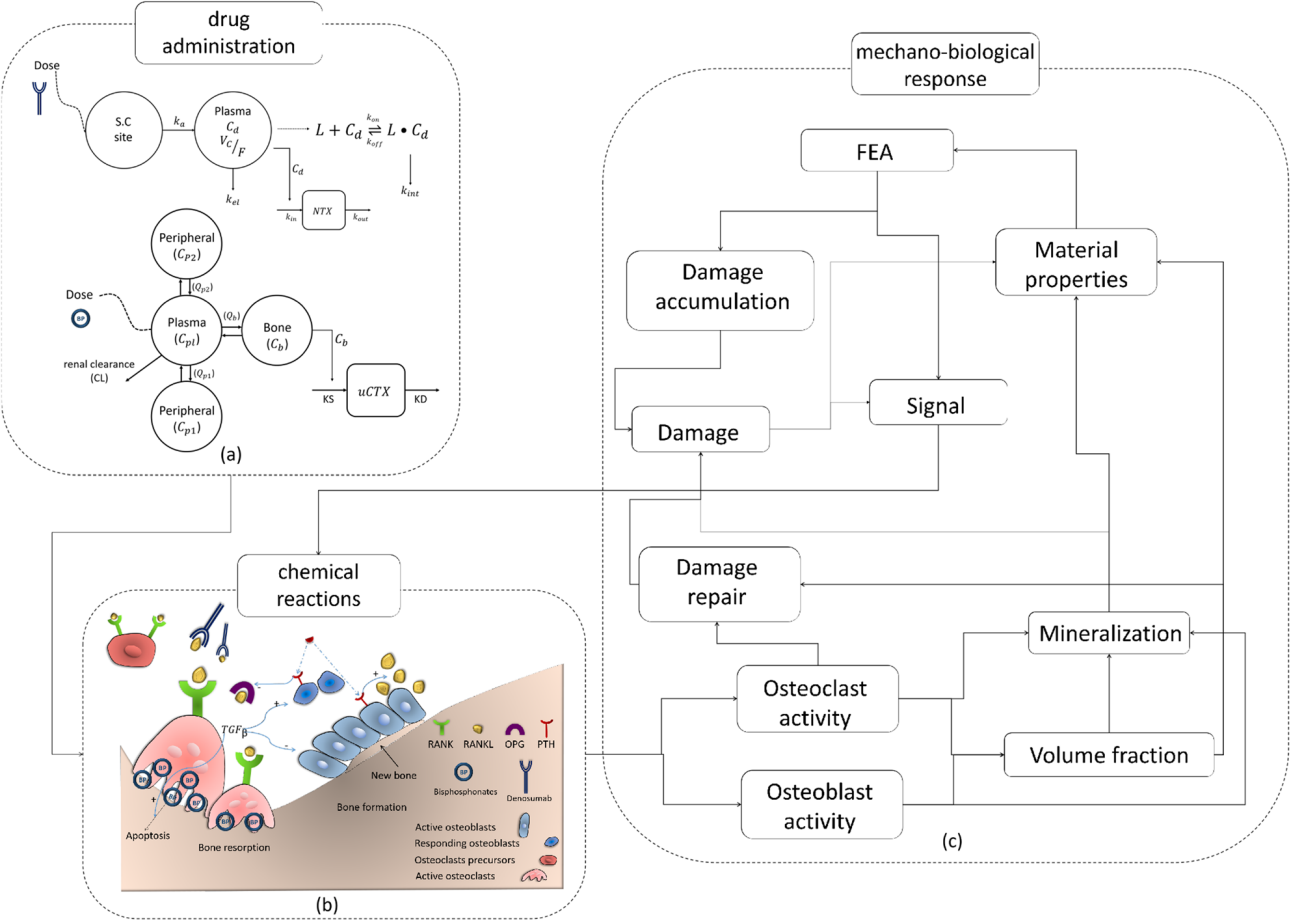
Schematic representation of the bone remodeling model coupled with drug administration: (**a**) PK/PD models for denosumab (top) and for Ibandronate (bottom); (**b**) effect of the biochemical pathway and bone cells; (**c**) flowchart of mechano-biological response bone remodeling model.

This model considers two compartments (subcutaneous and plasma) as shown in Fig. 11a. The first two equations determine the drug pharmacokinetics providing the drug concentration in the plasma compartment. In contrast, the third equation determines the pharmacodynamics in the action site, which reflects the maximal drug effect on the bone tissue. In these equations, *Dose* corresponds to the denosumab dose administrated subcutaneously during each time interval, $C_{tot}$ is the total drug concentration, sum of the free drug ($C_{d}$) and the drug–ligand complex ($L \bullet C_{d}$), $k_a$ is the first-order absorption rate of the drug administrated subcutaneously into the plasma compartment, $V_c/F$ is the bioavailability-adjusted central compartment volume, $k_{int}$ is the drug–ligand complex internalization or degradation, $k_{el}$ the drug removal rate from the central compartment, $R_{ss}$ the concentration of free ligand in the steady-state situation, $K_D=k_{off}/k_{on}$ the equilibrium disassociation constant for the drug–ligand reaction (Eq. (10)), *NTX* the crosslinked N-telopeptide of collagen type I, a bone turnover biomarker measured in the serum, which determines the drug response in bone and, finally, $k_{in}$ and $k_{out}$ are the production and removal rates of NTX, respectively. The steady-state values of *NTX* ($NTX_{ss}=k_{in}/k_{out}$) for multiple myeloma patients receiving 0.1, 0.3, 1.0 and 3.0 mg/kg of denosumab were chosen from^33,36^ who gave values of 9.8, 9.1, 10.2 and 8.1 nM respectively.

Similarly, the PK/PD model for bisphosphonates as Ibandronate may be written as^35^:

12 $$\begin{aligned} \begin{aligned} \frac{dC_{pl}}{dt}&=-\frac{(CL+Q_{p1}+Q_{p2}+Q_{b})}{V_{pl}}\times C_{pl} +\frac{Q_{p1}}{V_{p1}}\times C_{p1}+\frac{Q_{p2}}{V_{p2}}\times C_{p2}+\frac{Q_{b}}{V_{b}}\times C_{b}, \ C_{pl}(0)=Dose\\ \frac{dC_{p1}}{dt}&=\frac{Q_{p1}}{V_{pl}}\times C_{pl}-\frac{Q_{p1}}{V_{p1}}\times C_{p1}, \ C_{p1}(0)=0 \\ \frac{dC_{p2}}{dt}&=\frac{Q_{p2}}{V_{pl}}\times C_{pl}-\frac{Q_{p2}}{V_{p2}}\times C_{p2}, \ C_{p2}(0)=0 \\ \frac{dC_{b}}{dt}&=\frac{Q_{b}}{V_{pl}}\times C_{pl}-\frac{Q_{b}}{V_{b}}\times C_{b}, \ C_{b}(0)=0 \\ \frac{duCTX}{dt}&=KS\times (1+\frac{R_{tar}-KS}{KS}\times [1-e^{-k_{qq}t}])\times (1-\frac{(C_{b}/V_{b})^n}{{IC_{50}}^n+(C_{b}/V_{b})^n})-KD \times uCTX \end{aligned} \end{aligned}$$

The first four equations establish the four-compartment pharmacokinetic model for bisphosphonates^35^, providing the Ibandronate concentration in plasma, bone and two peripheral compartments (see Fig. 11a). In these equations, the subscripts *pl*, *p*1, *p*2 and *b* stand for plasma, peripheral1, peripheral2 and bone compartments, respectively. $C_{i}(i=pl,\ p1,\ p2,\ b)$ is the drug concentration in the compartment *i*, $Q_{i}(i=p1,\ p2,\ b)$ is the inter-compartmental clearance, $V_{i}(i=pl,\ p1,\ p2,\ b)$ the volume of each compartment, and *CL* the renal clearance. *Dose* corresponds to the Ibandronate dose administrated intravenously during each time interval and $C_{pl}$ is the sum of current drug concentration and the amount administrated at the beginning of the dosage interval. The fifth equation shows the pharmacodynamics of Ibandronate in bone, with *uCTX* the Urinary C-Terminal Telopeptide of Collagen Type I, a bone turnover biomarker measured in urine, *KS* and *KD* the *uCTX* formation and degradation rates, respectively, $R_{tar}$ the limit value for the *uCTX* formation rate defined by the rate constant of $k_{qq}$, $IC_{50}$ the Ibandronate concentration in the bone compartment producing 50% of maximum response of *uCTX* and *n* the Hill coefficient^35^. The PK/PD parameters used in this study are shown in Table 2, with the corresponding references.

**Table 2:** Values and description of the mechanical parameters.

	Description	Unit	Value	**References**
**Denosumab**				
Dose	Drug dose	mg/kg	0.1, 0.3, 1.0, 3.0	^33^
$k_a$	Absorption rate	1/day	0.167	^33^
$k_{int}$	Drug–ligand complex internalization	1/day	$2.67 \times 10^{-2}$	^33^
$k_{el}$	Elimination rate of drug from central compartment	1/day	$2.12 \times 10^{-2}$	^33^
$K_{D}$	$K_D=k_{off}/k_{on}$	M	$3.0 \times 10^{-12}$	^33^
$V_C/F$	Central compartment volume	l/kg	0.114	^33^
$R_{ss}$	Steady-state free ligand concentration	nM	1.07	^33^
$I_{max}$	Maximal fractional extent of inhibition	–	0.331	^33^
$IC_{50}$	Concentration producing 50$\%$ of maximal inhibition	nM	2.64	^33^
$k_{out}$	Rate of loss of response	1/day	0.572	^33^
**Ibandronate**				
Dose	Drug dose	mg	0.25, 0.5, 1.0, 2.0	^8^
$V_{pl}$	Plasma compartment volume	l	4.30	^35^
$V_{p1}$	Peripheral-1 compartment volume	l	2.80	^35^
$V_{p2}$	Peripheral-2 compartment volume	l	8.70	^35^
$V_{b}$	Bone compartment volume	l	609.00	^35^
$Q_{p1}$	Plasma-peripheral-1 compartmental clearances	l/day	69.43	^35^
$V_{p2}$	Plasma-peripheral-2 compartmental clearances	l/day	18.57	^35^
$V_{b}$	Plasma-bone compartmental clearances	l/day	51.71	^35^
*CL*	Renal clearance	l/day	57.00	^35^
*KS*	*uCTX* formation rate	$\mu \hbox {g mmol CR}^{-1}\hbox {day}^{-1}$	231.43	^35^
*KD*	*uCTX* degradation rate	1/day	0.68	^35^
$R_{tar}$	Limiting value of *uCTX* formation rate	$\mu \hbox {g mmol CR}^{-1}\hbox {day}^{-1}$	194.29	^35^
$k_{qq}$	Rate constant by which $R_{tar}$ obtained	l/day	0.0024	^35^
$IC_{50}$	Ibandronate concentration producing 50% of maximum response	$\mu g l^{-1}$	0.37	^35^
*n*	Hill coefficient	–	1.92	^35^

### Mineralization

Newly created bone tissue is mainly composed of collagen osteoid that is, then, progressively mineralized. Mineralization is generally divided in two phases: the first one is fast (it lasts only several days) reaching about 60$\%$ of the maximum mineral content in mature bone, while the second is much slower, lasting several years to achieve the final mineral content in fully developed bone^55,57^. Here, as in our previous model^34^, and in^55^, the first phase was assumed as instantaneous, following bone mineralization an exponential evolution during the second stage, that is:

13 $$\begin{aligned} \alpha \left( t\right) ={\alpha }_{max}+\left( {\alpha }_0-{\alpha }_{max}\right) e^{-\kappa t} \end{aligned}$$

with ${\alpha }_{max}$ and ${\alpha }_0$ the maximum and initial ash fractions (bone residual after calcination that essentially corresponds to the mineral content), respectively, and $\kappa$ the exponential mineralization constant.

Taking this into account, and for a representative volumen element, the mean ash fraction at a certain time after periods of new bone formation and resorption may be written as^55^:

14 $$\begin{aligned} {\bar{\alpha }}(t)=\frac{(v_{b,0}-h_0)\alpha (t)+\int _0^t{\left[ \left( \dot{v}_F(\tau ) -h(\tau )\right) \alpha (t-\tau )-\left( \dot{v}_R(\tau ) -h(\tau )\right) {{\overline{\alpha }}} (t-\tau ) \right] d\tau }}{v_b(t)-h(t)} \end{aligned}$$

with $v_{b,0}$ the initial bone volume fraction, while, from Eq. (1), ${\dot{v}}_F=\frac{dB_a}{dt}$ and ${\dot{v}}_R=\frac{dC}{dt}$ are the rates of bone volume fraction of newly created and resorbed bone, respectively and $h_0$ is the initial microcrack density defined as the number of microcracks per unit volume.

### Damage

Damage is here associated with the density of microcracks and strongly affects the mechanical properties of bone as well as the signaling process among cells. Here, the microcrack density, *h*, is assumed to have a linear relation with the damage level *d*, such as $h=kd$, with $k=0.00034$^55^.

Bone, as a living tissue, is able to repair those micro-cracks, so damage increases when having high stresses/strains, $\dot{d}_{acc}$ (damage accumulation rate), while, at the same time, microcracks are removed in regions where bone is resorbed, $\dot{d}_{rep}$ (damage repair rate)^2^. We can then write:

15 $$\begin{aligned} \dot{d}=\dot{d}_{acc}-\dot{d}_{rep} \end{aligned}$$

As stated in^34^ the damage accumulation for a certain number of cycles is a function of the load amplitude and the type of stress state (tension, $d_{acc,t}$, or compression, $d_{acc,c}$) can be written as:

16 $$\begin{aligned} \begin{aligned} d_{acc,c}&=-\frac{1}{\gamma _1}ln\left( 1-C_1{{\overline{\varepsilon }}}^{{\delta }_1}N\right) , \\ d_{acc,t}&=1-\root \gamma _2 \of {\frac{1}{C_3}ln\left( e^{C_3}-C_2{{\overline{\varepsilon }}}^{{\delta }_2}N\right) }, \\ {\delta }_1&=10.3,\ \ \gamma _1=-5.238\left( \left( {E}/{E^*}{\overline{\varepsilon }}-6100\right) +7\right) {10}^{-3},\ \ C_1=\frac{1-e^{{-\gamma }_1}}{9.333\times {10}^{40}}, \\ {\delta }_2&=14.1,\ \ \gamma _2 =-0.018\left( {E}/{E^*}{\overline{\varepsilon }}-4100\right) +12,\ \ C_2=\frac{e^{C_3}-1}{1.445\times {10}^{53}},\ \ C_3=-20, \end{aligned} \end{aligned}$$

which ${\delta }_{1,2}$, $\gamma _{1,2}$ and $C_{1,2,3}$ are parameters. *N* is the number of load cycles and ${\overline{\varepsilon }}=\sqrt{2u/E}$ is the equivalent strain in each of those cycles, which described before, with *u* the strain energy density and *E* the elastic modulus. $E^*$ is the the reference elastic modulus which for undamaged ($d=0$) cortical bone the ratio $E/E^*$ is equal to one.

Fatigue life ($N_f$) in compression and tension calculated as:

17 $$\begin{aligned} N_f=\frac{K_{i}}{{\overline{\varepsilon }} ^{ \delta _{i}}} = \left\{ \begin{array}{c}\frac{9.333 \times 10^{40}}{\frac{E}{E^*}{{\overline{\varepsilon }}}^{10.3}} \qquad \text {in compression}\\ \frac{1.445 \times 10^{53}}{\frac{E}{E^*}{{\overline{\varepsilon }}}^{14.1}} \qquad \text {in tension}\end{array}\right. , i=c \left( compression \right) ,t \left( tension \right) \end{aligned}$$

Finally, a Miner rule^59^ was used the determine the increase in damage for a certain number of cycles, using the bone fatigue life ($N_{f}$) for each strain level and for a particular bone calcium content as stated by Martinez et al.^57^, being this latter directly related with the ash fraction as $\left[ Ca \right] =\frac{259.2}{0.69} \alpha$^60^. The relationship between $K_{t}$ and the amount of calcium ($\left[ Ca \right]$) in the bone is expressed by the following equations:

18 $$\begin{aligned} K_{t} \left( \left[ Ca \right] \right) =10^{7} \left( \frac{ \varepsilon _{u} \left( \left[ Ca \right] \right) }{ \beta } \right) ^{ \delta _{t}} \end{aligned}$$

where $\delta _{i}$ and $\beta$ are constants. $\varepsilon _{u}$ is ultimate strain which has relation with calcium content:

19 $$\begin{aligned} log \varepsilon _{u}=25.425-11.341log \left[ Ca \right] \end{aligned}$$

Finally damage repair evolution is calculated as follows:

20 $$\begin{aligned} {{\dot{d}}}_{rep}={{\dot{v}}}_R\frac{d}{v_b}, \end{aligned}$$

where ${\dot{v}}_R=k_{res}C$ is the rate of bone volume fraction due to osteoclasts activity.

### Mechanical properties

Finally, as a first approach, and despite the well-known local orthotropy of bone tissue^61^, we assumed bone tissue as heterogeneous and isotropic with its mechanical properties defined by the following correlation between the volume fraction ($v_b$), ash fraction ($\alpha$) and damage (*d*), with the bone elastic modulus^55,62^:

21 $$\begin{aligned} E=84370v^{2.58}_b{\alpha }^{2.74}\left( 1-d\right) \end{aligned}$$

A summary of the mechanical parameters used in these study are presented in Table 3.

**Table 3:** Values and description of the mechanical parameters.

	Description	Unit	Value	References
*N*	Number of cycles	–	10000 (500 for mandible)	^55,63,64^
*m*	Weighing exponent	–	4	^55,56,65^
${\xi }^*_0$	Reference equilibrium stimulus		0.0025	^55^
*c*	Stimulus activation curve parameter	–	0.0025	^55^
*a*	Damage activation curve parameter	–	20	–
$d_0$	Initial damage	–	0	^55^
${\alpha }_{ini}$	Initial ash fraction	–	0.6	^55,65,66^
${\alpha }_0$	Minimal ash fraction	–	0.45	^55,66,67^
${\alpha }_{max}$	Maximum ash fraction	–	0.7	^55,66,67^
$\kappa$	Secondary mineralization period	years	6	^55^
$\beta$	Fatigue limit coefficient	–	5	–

Finally, a scheme of the chemo-mechano-biological bone remodeling model, coupled with the PK/PD models for the two drug contents, is illustrated in Fig. 11.

### Finite element simulation

**Figure 12:**
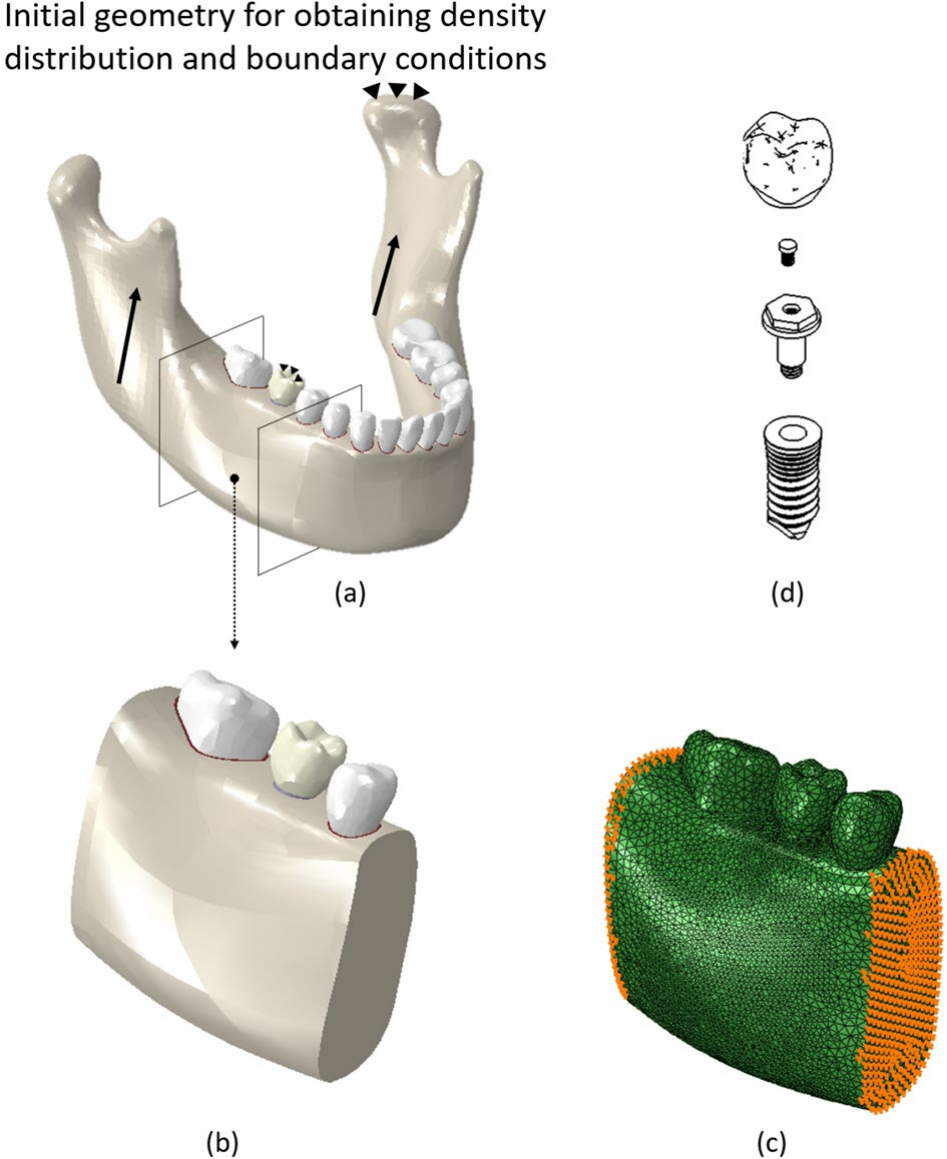
(**a**) Mandible geometry. The arrows represent the muscle reaction forces while the triangles represent the boundary condition for the case of mastication with the implant (first right molar); (**b**) cut part from the complete geometry to study the drug effect on osteoporotic bone; (**c**) finite element model of the cut part. Displacement boundary conditions are applied onto the mesial and distal surfaces, and obtained from the initial simulation with the complete mandible; (**d**) components of the implant and crown.

Computed tomography images of a healthy adult woman were used to construct the 3D geometric model of the mandible. After segmenting the mandible and teeth in MIMICS 10 (Materialise, Leuven, Belgium) and obtaining the STL files, CATIA (CATIA V5, Dassault Systèmes, Vèlizy-Villacoublay, France) was used to create the final three-dimensional geometry of the mandible, teeth, and PDLs (see Fig. 12). The gaps between the mandible and the teeth were used to obtain the geometry of the PDLs. The implant selected for this analysis is based on the INTRI design without internal resilient parts^68^. The height of the implant was 11 mm and its diameters at the top and bottom were 5.1 mm and 4.5 mm with two threaded steps of 1 and 0.5 mm respectively. Finally, the crown was designed for the first right molar, taking into account the implant neck.

The model here developed is based on physiological mechanisms and properties, so, contrary to other phenomenological bone remodeling models, it leads to wrong results when the initial density distribution is not physiological and related to the initial values of the cell concentrations. Therefore, the geometry in Fig. 12 was used first to obtain the initial density distribution for the next simulations. A phenomenological bone remodeling model^56,69^ was used for this purpose, considering the mastication muscles’ reaction forces, while the boundary conditions were applied to each teeth involved in the mastication process based on previous studies^63,70^. After simulation of the complete mandible (540 simulation steps), we correlated such initial density distribution with the elastic modulus point-wise, using the following correlations: $E=1736\rho ^{3.2}$ and $E=2014\rho ^{2.5}$ for cortical and trabecular bone, respectively^63^. Finally, those values were modified to take into account osteoporosis. Reductions of 33% 66% have been reported for the modulus of elasticity for cortical ($\rho >1.2g/cc$) and trabecular bones ($\rho <1.2g/cc$), respectively, in osteoporotic patients^17^. Therefore, we modified the initial distribution of the elastic moduli in each of the bone types considering such values.

To reduce the computer time, a cut of bone, which includes the premolar tooth and its PDL, the second molar and its PDL and the implant, was isolated to perform the next simulations. The whole geometric model, together with the density distribution (and corresponding material properties), was then exported to ABAQUS (ABAQUS 6.11, Dassault Systèmes, Vèlizy-Villacoublay, France) to perform the finite element simulations. A four-noded solid tetrahedral mesh was built in ABAQUS-CAE. The final number of elements was obtained after ensuring sufficient accuracy in a previous convergence analysis. The final resulting number of elements for the whole mandible, teeth, PDLs, implant, and crown in the final model was 860064, 233083, 30369, 46939 and 5325, respectively, while the number of elements in the model of the section cut for bone, teeth, and PDLs was 448265, 12896 and 4729 respectively.

Complete osseointegration was assumed for the bone-implant, bone-PDL and PDL-tooth interfaces. Displacement at the nodes of the mesial and distal surfaces of the cut model was imposed with values derived from the results of the complete mandible model (Fig. 12). The Titanium implant and crown materials were assumed as linearly elastic with $E=118 \,\hbox {GPa}, \ \nu =0.35$ and $E=82.8 \,\hbox {GPa}, \ \nu =0.33$ respectively^71,72^. Finally, the bone mechanical properties change during the bone remodeling process, so a user material (UMAT) subroutine of ABAQUS was implemented to compute such properties along the loading process according to the model described above. To simulate the effect of drug treatment in the bone surrounding the dental implant, different drug doses were used and the corresponding results compared with the control model without the drug.
